# Fusobacterium Bacteremia Causing Hepatic Abscess in a Patient With Diverticulitis

**DOI:** 10.7759/cureus.26938

**Published:** 2022-07-17

**Authors:** Aditi Bawa, Aleesha Kainat, Haseeb Raza, Tissa B George, Hanan Omer, Anjana C Pillai

**Affiliations:** 1 Internal Medicine, University of Pittsburgh Medical Center (UPMC) McKeesport, Pittsburgh, USA; 2 Internal Medicine, Gujranwala Medical College, Gujranwala, PAK

**Keywords:** hepatic abscess, left sided abdominal pain, complicated diverticulitis, fusobacterium nucelatum, pyogenic hepatic abscess

## Abstract

Fusobacterium, a gram-negative non-spore-forming anaerobe, is a common inhabitant of the oral cavity; however, it is not typically found in other organ systems. The occurrence of a hepatic abscess associated with this organism is rare. We present a patient with recurrent diverticulitis with left upper quadrant abdominal pain, and abdominal imaging revealed a hepatic abscess in addition to sigmoid diverticulitis. Further investigations led to an unusual culprit; Fusobacterium nucleatum.

## Introduction

Acute diverticulitis (AD) is a common cause of abdominal pain, and additional symptoms include fever, abdominal distension, diarrhea, constipation, and nausea. Complicated acute diverticulitis is associated with higher mortality; therefore, it is essential to be aware of its complications and diagnose them promptly. Complications include abscess formation, diverticular hemorrhage, fistula formation, bowel obstruction, sepsis, and septic shock. CT is commonly performed to diagnose diverticulitis radiographically and establish the occurrence of associated complications. Pyogenic liver abscess, the most common intra-abdominal abscess, is rarely associated with acute diverticulitis, and it can commonly co-occur with portal vein pyemia, peritonitis, or biliary tract infection [1,2]. Enteric-gram negative bacilli and streptococci are most commonly cultured in pyogenic liver abscesses; however, anaerobes are likely under-reported due to difficulty in culturing and laboratory characterization.

## Case presentation

**Figure 1:**
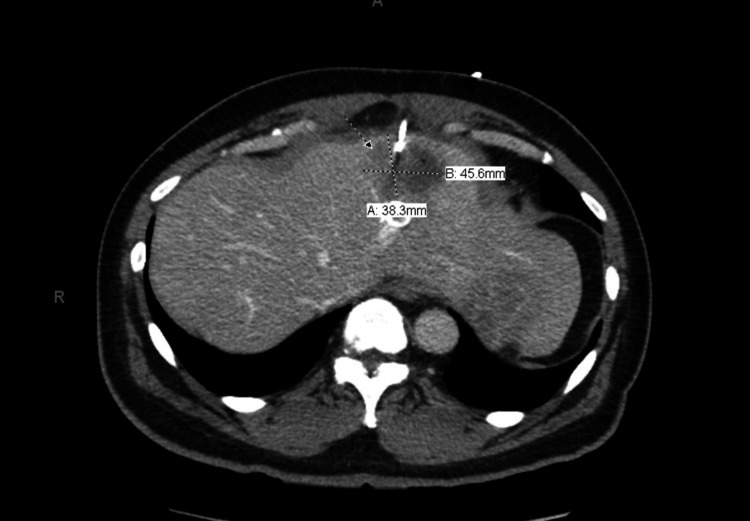
Arrow point towards hepatic abscess with percutaneous drainage in place

A 48-year-old male with a medical history notable for chronic constipation and recurrent diverticulitis presents to the hospital with a chief complaint of left upper quadrant (LUQ) pain, fever, and chills. The patient was in his usual state of health until a day prior when he started to develop LUQ pain which worsened overnight, prompting him to come to the hospital the following day. The pain was described as a stabbing sensation, non-radiating, and continuous. Of note, the patient was recently treated for acute diverticulitis with antibiotics (Ciprofloxacin and Metronidazole) two months prior to the hospital visit. Vitals on presentation; temperature 102.8F, blood pressure 166/88 mmHg, heart rate 102, respiratory rate 30 breaths per minute, saturating 98% on room air. Abdominal examination revealed left upper and lower quadrant pain on palpation. Labs on presentation are displayed in table 1. CT abdomen and pelvis revealed a loculated 4.2x3.8cm hypodense mass in segment 2 of the left hepatic lobe concerning hepatic abscess and pan colonic diverticulosis with acute diverticulitis of sigmoid/descending colonic junction and mid ascending colon (Figure 1). The patient was initiated on intravenous (IV) piperacillin-tazobactam.

**Table 1:** Lab work on presentation BUN- blood urea nitrogen; ALT- alanine transaminase; AST- aspartate transaminase; PT- prothrombin time; PTT- Partial thromboplastin time; INR- international normalized ratio

Labs	Patients values	Normal reference range
Sodium	139	136-145 mEq/L
Potassium	3.7	3.5-4.9 mEq/L
Chloride	109	98-109 mEq/L
Anion Gap	9.7	7-15 mEq/L
BUN	14	7-22 mg/dL
Creatinine	1.2 (near baseline)	0.6-1.6 mg/dL
Ca	8.3	8.3-10.5 mg/dL
Albumin	3.2	3.4-5 gm/dL
Total Bilirubin	0.2	0.1-1 mg/dL
ALT	47	1-65 Units/L
AST	36	1-37 Units/L
Alkaline Phosphatase	100	39-119 Units/L
Lactate	1.2	0.5-2.2 mMol/L
Lipase	128	73-393 Units/L
WBC	10.5	3.8-10.6 X10E+09/L
Neutrophils %	82.8%	44.3-77.1%
Hemoglobin	11.1 (near baseline)	12.9-16.9 gm/dL
PT	12.4	11.8-14.4 seconds
PTT	33.5	23-33.8 seconds
INR	1	0.8-1.2
Troponin	0.030	<0.045 ng/mL

Surgery and Infectious diseases were consulted. Per their recommendations, Interventional Radiology (IR) was involved in the percutaneous ultrasound-guided drainage of the abscess and gram stain/culture of the aspirated fluid. Initial drainage was blood-tinged; subsequently, dark, bloody fluid was noted. The aspirate was sent for gram stain and culture. The patient was maintained on IV antibiotics. Within a few days, the aspirate fluid grew Fusobacterium, and blood cultures were positive 2/4 anaerobic bottles for Fusobacterium nucleatum. 

The patient received IV antibiotics (Zosyn) throughout hospitalization with improved clinical symptoms. The patient was discharged on IV antibiotics. Serial CT scans were performed as an outpatient to monitor changes in the hepatic lesion. Repeat CT scan approximately one month after discharge showed near-complete resolution of the left lobe liver abscess with associated granulation. Antibiotics were deescalated to oral Augmentin for two weeks. An additional CT 2 months after demonstrated complete resolution of the hepatic abscess.

## Discussion

Fusobacterium, a gram-negative anaerobe, is a rare cause of hepatic abscess [3]. Bacteremia caused by Fusobacterium is less than 1% cause of anaerobic bacteremia [4]. Our case is unique because our patient presented with left upper quadrant pain, unlike right upper quadrant (RUQ) pain which is more common in pyogenic liver abscesses. It can be hypothesized that since our patient had a liver abscess in the left lobe of the liver, it may have led to LUQ abdominal pain rather than the classic RUQ abdominal pain seen with liver abscesses. Furthermore, the patient was empirically managed for acute diverticulitis with a low clinical suspicion for associated complications due to the history of recurrent diverticulitis. Imaging was the only reason for early identification and management of the hepatic lesion. 

Mortality associated with hepatic abscess is estimated to be around 15% [5]. This is attributed to the inability to diagnose and manage timely. Per specific guidelines, the recommendation for workup of a pyogenic abscess includes obtaining cultures from the lesion along with blood cultures to tailor specific antibiotic therapy [6]. In addition to antibiotic therapy, some cases require percutaneous or surgical drainage for source control. 

Our patient underwent percutaneous ultrasound-guided drainage of the hepatic lesion, and the aspirate culture grew Fusobacterium nucleatum. Fusobacterium nucleatum has been associated with many GI disorders such as appendicitis, colorectal cancer, and inflammatory bowel disease [1], but there is little data on its association with hepatic lesions. Given the presence of this organism in the oral cavity, the likely pathogenesis involves translocation of the pathogen from the gastrointestinal tract to the liver by portal circulation or after a micro-perforation of the inflamed diverticula leading to bacteremia.

## Conclusions

Pyogenic liver abscess is a life-threatening complication of diverticulitis. Reduction in mortality and morbidity is possible with early recognition and management. Physicians must remember that patients with recurrent diverticulitis can develop rare complications such as a pyogenic liver abscess. Uncommon causes of hepatic abscesses require specific antibiotic therapy; therefore, determination of the causative agent becomes key in effective management.
